# Silicon Uptake and Localisation in Date Palm (*Phoenix dactylifera*) – A Unique Association With Sclerenchyma

**DOI:** 10.3389/fpls.2019.00988

**Published:** 2019-08-13

**Authors:** Boris Bokor, Milan Soukup, Marek Vaculík, Peter Vd’ačný, Marieluise Weidinger, Irene Lichtscheidl, Silvia Vávrová, Katarína Šoltys, Humira Sonah, Rupesh Deshmukh, Richard R. Bélanger, Philip J. White, Hamed A. El-Serehy, Alexander Lux

**Affiliations:** ^1^Department of Plant Physiology, Faculty of Natural Sciences, Comenius University in Bratislava, Bratislava, Slovakia; ^2^Comenius University Science Park, Bratislava, Slovakia; ^3^Institute of Chemistry, Slovak Academy of Sciences, Bratislava, Slovakia; ^4^Institute of Botany, Plant Science and Biodiversity Centre, Slovak Academy of Sciences, Bratislava, Slovakia; ^5^Department of Zoology, Faculty of Natural Sciences, Comenius University in Bratislava, Bratislava, Slovakia; ^6^Core Facility of Cell Imaging and Ultrastructure Research, University of Vienna, Vienna, Austria; ^7^Department of Molecular Biology, Faculty of Natural Sciences, Comenius University in Bratislava, Bratislava, Slovakia; ^8^Department of Plant Science, Université Laval, Quebec, QC, Canada; ^9^The James Hutton Institute, Dundee, United Kingdom; ^10^Distinguished Scientist Fellowship Program, King Saud University, Riyadh, Saudi Arabia; ^11^Zoology Department, College of Science, King Saud University, Riyadh, Saudi Arabia

**Keywords:** Arecaceae, cell wall composition, date palm (*Phoenix dactylifera*), phylogenetic analysis, phytoliths, plant anatomy, silicon (Si) transporters, stegmata

## Abstract

Date palm (*Phoenix dactylifera*) can accumulate as much as 1% silicon (Si), but not much is known about the mechanisms inherent to this process. Here, we investigated in detail the uptake, accumulation and distribution of Si in date palms, and the phylogeny of Si transporter genes in plants. We characterized the PdNIP2 transporter following heterologous expression in *Xenopus* oocytes and used qPCR to determine the relative expression of Si transporter genes. Silicon accumulation and distribution was investigated by light microscopy, scanning electron microscopy coupled with X-ray microanalysis and Raman microspectroscopy. We proved that *PdNIP2-1* codes for a functional Si-permeable protein and demonstrated that *PdNIP2* transporter genes were constitutively expressed in date palm. Silicon aggregates/phytoliths were found in specific stegmata cells present in roots, stems and leaves and their surfaces were composed of pure silica. Stegmata were organized on the outer surface of the sclerenchyma bundles or associated with the sclerenchyma of the vascular bundles. Phylogenetic analysis clustered NIP2 transporters of the Arecaceae in a sister position to those of the Poaceae. It is suggested, that Si uptake in date palm is mediated by a constitutively expressed Si influx transporter and accumulated as Si aggregates in stegmata cells abundant in the outer surface of the sclerenchyma bundles (fibers).

## Introduction

Silicon (Si) is not considered to be an essential element for plants, but its tissue concentration can exceed that of many essential elements in some plant species (Hodson et al., 2005; White and Brown, 2010). The roles of Si as a beneficial element for plants, protecting them from a variety of abiotic stresses and biotic challenges, have been discussed in the literature for a long time (Epstein, 1999; Coskun et al., 2018).

In most circumstances, plant roots take up Si from the soil solution and it is then transported to the aboveground organs via the xylem (Casey et al., 2003; Mitani et al., 2005). The accumulation of Si varies greatly among plant species and those belonging to the commelinid monocot orders Poales (e.g., cereals, grasses, bromeliads, and sedges) and Arecales (e.g., palms) generally accumulate more Si than other plants (Hodson et al., 2005). The identification of genes encoding proteins responsible for Si transport have shown that Si accumulation is the result of an efficient symplastic pathway mediated by Si influx and efflux transport mechanisms in the plasma membrane of root cells (Ma and Yamaji, 2015). Silicon influx proteins, termed Lsi1, are members of the NIP III (nodulin 26-like intrinsic protein III) group of aquaporin-like proteins belonging to the large MIP (major intrinsic protein) superfamily that contains various classes of integral membrane proteins functioning as diffusion facilitators of water and small uncharged solutes (Wallace and Roberts, 2005; Ma and Yamaji, 2015; Pommerrenig et al., 2015; Deshmukh et al., 2016). Aquaporins in the NIP III group contain two hallmark domains: a unique selectivity filter (ar/R filter, also known as GSGR filter) formed by glycine (G), serine (S), glycine (G) and arginine (R) and two NPA motifs (also referred to as NPA boxes) consisting of asparagine (N), proline (P) and alanine (A) separated by 108 amino acids (Deshmukh et al., 2015; Ma and Yamaji, 2015).

Following its uptake by roots, Si can be deposited in plant tissues in various forms, most frequently in silica cells or silica bodies distributed within the leaf epidermis or as a dense layer beneath the cuticle (Datnoff et al., 2001; Coskun et al., 2018). Other common sites of Si deposition are specialized cells termed stegmata that form a sheath around sclerenchyma fibers attached to vascular bundles or individual fiber bundles in species such as palms in the commelinid monocot order Arecales and orchids in non-commelinid monocot order Asparagales (Møller and Rasmussen, 1984). Root tissues are also sites of Si accumulation in some plant species, with the endodermis being the dominant deposition site, especially in monocots (Sangster and Hodson, 1992; Lux et al., 2003).

Silicon deposition in palms is a well-known, but poorly understood phenomenon. This study is focused on a detailed description of date palm anatomy as it relates to the unique Si distribution in this species and presents novel observations on Si uptake mechanisms in date palms and the phylogenetic relationships between the Si transport proteins of date palms and other Si-accumulating species.

## Materials and Methods

### Plant Cultivation

In our studies, we compared three developmental stages of date palms: young seedlings (ca 1-month-old) grown in hydroponics, 1-year-old plants grown in perlite, and 10-year-old plants grown in soil. For the oocyte experiments, RNA was extracted from roots of 1-week-old date palm and rice plants grown in hydroponics.

Prior to cultivation in hydroponics and perlite, date palm seeds were surface sterilized in 2.5% NaClO solution for 10 min and washed several times with dH_2_O. After such treatment, germination took about 2.5 weeks. In hydroponics, two different treatments were imposed: a Si- control treatment with Hoagland solution (Hoagland and Arnon, 1950) and without silicon supplementation, and an Si+ treatment with Hoagland solution and Si addition as sodium silicate [Na_2_O(SiO_2_)_x_.xH_2_O, or given also as Na_2_O_7_Si_3_ by Sigma-Aldrich] to a final concentration of 1 mM (this compound is referred as Si in the text), or 0.084 g/L of elemental silicon. This Si concentration was chosen because it is similar to the Si concentrations in soil solutions and is recommended for laboratory studies (Epstein, 1994; Liang et al., 2015). Plants were grown in a growth chamber with 12 h light/12 h dark, a light intensity of 200 μmol PAR m^-2^ s^-1^, relative humidity of approximately 75% and day/night temperatures of 28/24°C. During the first 5 days, germinated plants were acclimatized to hydroponics by growing them in a half-strength Hoagland solution without Si addition. Subsequently, the two different treatments (Si- and Si+) were initiated and five plants were cultivated in 3 L pots for 21 days. Hoagland solution was renewed every third day and pH was adjusted to a value of 6.2.

Cultivation in 1L pots filled with perlite (68–73% SiO_2_, 7.5–15.0% Al_2_O_3_, 1.0–2.0% Fe_2_O_3_, 0.5–2.0% CaO, 0.2–1.0% MgO, 2.0–5.5% K_2_O, 2.5–5.0% Na_2_O, max. 1.0% TiO_2_, max. 0.2% P_2_O_5_, max. 0.3% MnO) lasted about 12 months. Plants (one per pot) were watered once a week with half strength Hoagland solution (200 mL). Plants were grown in a growth chamber with conditions identical to hydroponically cultivated plants.

In addition, 10-year-old plants grown in soil in the greenhouse at the Department of Plant Physiology, Faculty of Natural Sciences, Comenius University in Bratislava, were studied. Plants were watered regularly with tap water and every second year they were transferred to a bigger pot containing fresh sandy-loam soil with a bioavailable Si concentration of 113 ± 15 mg kg^-1^ as described by Bokor et al. (2017). The final volume of the pot at the end of the cultivation was 60 L.

### Light Microscopy

Hand sections were prepared as described by Lux et al. (2015). Cross and longitudinal sections of all organs studied, primary, lateral and adventitious roots, stem, shoot apex, leaf petioles, leaf sheaths, and leaf blades, were examined under a microscope (Axioskop 2 plus, Carl Zeiss, Germany) and documented using a digital camera DP72 (Olympus, Japan).

For general anatomy, both unstained sections and sections cleared with lactic acid and stained in an aqueous 0.05% (w/v) solution of toluidine blue were used. The Wiesner phloroglucinol-HCl reaction was used to identify lignification of cell walls in individual tissues. Suberin was visualized in sections cleared and stained with a 0.01% (w/v) solution of Fluorol Yellow 088 (FY088; Sigma-Aldrich) in lactic acid at 70°C for 1 h (Lux et al., 2015) and examined under an epifluorescence microscope (Axioskop 2 plus, Carl Zeiss, Germany; filter set Carl Zeiss N. 25: excitation filter TBP 400 nm + 495 nm + 570 nm, chromatic beam splitter TFT 410 nm + 505 nm + 585 nm, and emission filter TBP 460 nm + 530 nm + 610 nm).

Serial cross and longitudinal sections, of fixed, paraffin embedded and stained sections were used for additional studies of all organs. Briefly, the samples of individual organs were fixed in formalin–acetic acid–alcohol (FAA), dehydrated in a graded ethanol series, transferred to xylene and embedded in paraffin (Johansen, 1940). Sections, 15–20 μm thick, were deparaffinised in xylene and stained with alcian blue/safranin and mounted in Canada balsam. Observation and documentation were performed as described above.

### Scanning Electron Microscopy (SEM) Coupled With X-Ray Microanalysis

Transversely and longitudinally sectioned and air-dried root, stem and leaf tissues were fixed on aluminum stubs covered with a carbon sticker. Surface conductivity was increased by carbon coating, which in turn also resulted in a uniform, approximately 60 nm thick, carbon layer on the tissue surface. The distribution of Si was analyzed with a Jeol JSM-IT300 scanning electron microscope (SEM) equipped with an energy dispersive X-ray (EDX) analyser (EDAX, Octane Plus, Ametek, United States).

Plant phytoliths were examined at several different spots on each of the three plant tissues studied (root, stem, and leaf). Raw data were processed with the TEAM Enhanced ver. 4.3 (EDAX-Ametek, United States) software and all values were expressed as weight % of the total analyzed Si element.

### Total Si Concentrations in Plant Tissues

At the end of cultivation, the total Si concentration was measured in roots and second fully developed leaves of plants cultivated in perlite; and in roots, shoot apexes, leaf petioles, and leaf blades of plants cultivated in soil. The concentration of Si in the dry biomass of plant samples was determined using atomic absorption spectroscopy (AAS). Plant samples were dried at room temperature and ground to small pieces (<1 mm) with a mortar and a pestle. Digestions of plant samples were carried out in stainless steel coated PTFE pressure vessels ZA-1 (Czechia) in an electric oven at 160°C for 6 h. Each vessel contained between 0.1 and 0.5 g dried plant sample, 5 ml of concentrated HNO_3_, 0.25 ml of concentrated HF and 2 ml of 30% H_2_O_2_. After digestion, 2 ml of a saturated solution of H_3_BO_3_ was added and the resulting mixture was diluted to 25 ml with redistilled water and stored in a 100-ml polyethylene bottle. Silicon concentrations were determined by a flame atomic absorption spectrometry (AAS Perkin Elmer Model 5000, wavelength 251.6 nm, flame: acetylene-N_2_O). The concentration of bioavailable Si from the perlite (70 ± 5 mg kg^-1^) was analyzed according to Rodrigues et al. (2003) with appropriate modifications. After extraction by 0.5 M acetic acid, Si was measured by ICP-MS in place of colorimetric determination using blue silicomolybdous acid procedure as used in the original procedure, and as a quality control certified reference material for Si was analyzed, too. Analyses were performed at a certified laboratory of the Institute of Laboratory Research on Geomaterials (Faculty of Natural Sciences, Comenius University in Bratislava).

### Isolation of Silica Phytoliths

Hand cross-sections from the basal part of the leaf sheath were placed on a microscope slide and a drop of 96% sulfuric acid was added. After 5 min, several drops of distilled water were added, the sample covered with a cover slip and gently pressed to break the digested tissues. The isolated phytolith samples were then used either for dark field light microscopy or for Raman analyses.

### Raman Microspectroscopy

For Raman analyses, 15 μm thick microtome sections of paraffin embedded samples were prepared, dewaxed with 100% xylene for 30 min (2×) and gradually rehydrated in 20-min steps. A gradual series of mixtures of ethanol and distilled water was used (1:0; 1:0; 0.7:0.3; 0.5:0.5; 0.3:0.7; 0:1; 0:1). Sections were placed on microscope slides, mounted in distilled water, covered with coverslips and sealed with nail polish to avoid water evaporation. Hydrated silica gel was prepared as aqueous suspension of chromatography grade silica gel. Raman spectra were collected with a DXR Raman Microscope (Thermo Fisher Scientific, United States), equipped with a 532 nm laser, using 900 lines mm^-1^ grating. Spectra were recorded using 9 mW laser power, 12 s photobleaching time, with 10–30 s acquisition time per collection and eight collections per measurement. At least five spectra per structure were collected and analyzed. Omnic Atlas software (Thermo Fisher Scientific, United States) was used to collect the spectra. Spectral processing was performed using Spectragryph 1.0.7 (F. Menges “Spectragryph – optical spectroscopy software,” Version 1.0.7, 2017^1^). Spectra were baseline-corrected, smoothed (Sawitzky-Golay, 9 points, polynomial order 4) and normalized against a peak at 2895 cm^-1^ if not stated otherwise. Spectra are presented as means of all spectra collected from the object analyzed. The reference table used for peak assignments for these spectra are shown in Supplementary Tables S1, S2. The estimation of S/G-lignin ratio was based on the ratio of peak intensities 1334/1273 cm^-1^ (Lupoi and Smith, 2012). The estimation of cellulose crystallinity was based on the ratio of peak intensities 380/1096 cm^-1^ (Agarwal et al., 2010).

### RNA Extraction and cDNA Synthesis

On the third day of hydroponic cultivation and for the next 5 days, root tissues were sampled from plants growing in both Si- and Si+ treatments to evaluate gene expression. Samples (up to 150 mg) were stored at –80°C before RNA extraction. Total RNA was extracted and treated with DNase I using a Spectrum Plant Total RNA kit (Sigma–Aldrich, United States) according to the manufacturer’s instructions, except for the duration of DNase I treatment which was extended to 60 min. The RNA concentration and sample purity were measured using a NanoDrop^TM^ 1000 spectrophotometer (Thermo Fisher Scientific, Germany) and RNA integrity was checked by agarose (1%) gel electrophoresis. The synthesis of the first strand of cDNA was performed using an ImProm-II Reverse Transcription System (Promega, United States), using Oligo(dT)15 primers according to the manufacturer’s instructions. A control without RT was performed for each sample to determine whether there were any traces of genomic DNA. Samples containing only cDNA (10-times diluted) were used for qPCR analysis.

### Plasmid Constructions for Heterologous Expression in Xenopus Oocytes

The cDNA prepared from rice and date palm was used to amplify the open reading frames (ORF) of *OsLsi1* and *PdNIP2-1*. The ORFs amplified using Phusion Taq polymerase (New England Biolabs, Whitby, ON, Canada) were first cloned in a pUC18 plasmid vector and sequenced to confirm the accuracy of the ORFs. For heterologous expression in *Xenopus laevis* oocytes, the ORFs were further cloned using *Eco*RI/*Xba*I restriction sites into the Pol1 vector (PdNIP2-1EcoR1F: CCGAATTCATGGCTTCCTTTCCGAGAC, PdNIP2-1Xba1R: GTTCAATTGGAAAATGTTTGATCTAGAGC), a *X. laevis* oocyte expression vector derived from pGEM and comprising the T7 promoter, the *Xenopus* globin untranslated regions and a poly(A) tract (Caron et al., 2000). Both the plasmid constructs, OsLsi1-Pol1 and PdNIP2-1-Pol1, were transformed into *Escherichia coli* TOP10 strain and stored at -80°C. Correctness of the constructs was checked by sequencing (T7P: TAATACGACTCACTATAGG, Xeno3UTR: GACTCCATTCGGGTGTTCTTG) prior to *in vitro* translation.

### Si Transport Assays Using Heterologous Expression in *Xenopus* Oocytes

Plasmids containing either the *OsLsi1* or *PdNIP2-1* ORF were recovered from a fresh bacterial culture using a QIAprep Spin Miniprep kit (Qiagen^2^). Five micrograms of each plasmid was linearized using *Nhe*I (Roche^3^). Digested products were column-purified using a PCR purification kit (Qiagen), and 1 μg of plasmid DNA was transcribed *in vitro* using the mMessage mMachine T7 Ultra kit (Ambion^4^). Complementary RNAs (cRNAs) were purified using the lithium chloride precipitation method as described by the manufacturer and suspended in ultra-pure water.

The oocyte assays were performed as described by Deshmukh et al. (2013) with some minor changes. Oocytes at stage 5 or 6 were injected with 25 nl of 1 ng/nl cRNA or an equal volume of H_2_O as a negative control. Then oocytes were incubated for 1 day at 18°C in Barth’s (MBS) medium [88 mM NaCl, 1 mM KCI, 2.4 mM NaHCO_3_, 0.82 mM MgSO_4_, 0.33 mM Ca(NO_3_)_2_⋅4H_2_0, 0.41 mM CaCl_2_, 15 mM HEPES, pH 7.6] supplemented with 100 μM each of penicillin and streptomycin. Then, 10 sets of 10 oocytes for each condition were exposed to MBS solution containing 1.7 mM Si for 30 or 60 min. After exposure, oocytes were rinsed in solution containing 0.32 M sucrose and 5.0 mM HEPES (pH 7.4). Si quantification was performed with a Zeeman atomic spectrometer AA240Z (Varian, Palo Alto, CA, United States) equipped with a GTA120 Zeeman graphite tube atomizer. Data from the spectrometer were analyzed using JMP 9.0.2 (SAS Institute Inc.). Three replicates were used for this assay.

### Primer Design and RT-qPCR

In the NCBI database, two *PdNIP2* transcripts (mRNA sequences) with the following accession numbers XM_008804384.2 for *PdNIP2-1* and XM_008785804.2 for *PdNIP2-2* were available for date palm. The primers for the reference gene *actin* (XM_008778129.2) and *NIP2* genes (Supplementary Table S3) were designed using the Primer3plus tool^5^. Gradient PCR was performed to determine annealing temperature of primers. After that, PCR products were checked by agarose (2%) gel electrophoresis and sequenced by the Sanger method to verify product specificity at the Department of Molecular Biology, Faculty of Natural Sciences, Comenius University in Bratislava. Before qPCR analysis, the stability of the reference gene and efficiency of gene amplification was assessed (Livak and Schmittgen, 2001; Pfaffl et al., 2004). The reference gene, *PdNIP2-1* and *PdNIP2-2* genes were amplified by the Maxima SYBR Green/ROX qPCR Master Mix (Thermo Fisher Scientific, Germany) in 96-well plates using a Light Cycler II 480 (Roche, Switzerland). Melt curve analysis of amplification products was included at the end of each run of the qPCR reaction. The main purpose of the melt curve analysis was to check PCR product specificity; i.e., to confirm that only specific amplification and no non-specific PCR products or primer dimers were formed. The relative change in gene expression was estimated according to the Pfaffl method, including the amplification efficiency of the selected genes (Pfaffl, 2001).

### Bioinformatics and Statistics

Amino acid sequences were aligned using the MAFFT algorithm with one hundred bootstrap repeats on the GUIDANCE2Server^6^ (Sela et al., 2015). The confidence level of the resulting base multi sequence alignment (MSA) was estimated by comparing bootstrap trees as guide-trees to the alignment algorithm. Unreliably aligned columns were removed from the MSA at a cutoff value of 0.93. To analyze the effect of masking on tree inferences, all phylogenetic analyses were conducted also on the unmasked MSA.

Phylogenetic trees were constructed using both the Bayesian and the maximum likelihood techniques. Bayesian inference was performed using the computer program MrBayes ver. 3.2.6 (Ronquist et al., 2012) on the CIPRES Portal ver. 3.1^7^, using the WAG amino acid substitution model, four independent chains, one million generations and a sample frequency of one hundred. The first 25% of sampled trees were considered as burn-in and discarded. A 50% majority-rule consensus of the remaining trees was computed, and posterior probabilities of its branching pattern were estimated. Maximum likelihood analyses were performed using the computer program PhyML ver. 3.0 on the South of France bioinformatics platform^8^ (Guindon et al., 2010), with the SPR tree-rearrangement and 1000 non-parametric bootstrap replicates. The best amino acid substitution model for maximum likelihood analyses was selected automatically, using the Akaike Information Criterion as implemented in PhyML. Bayesian and maximum likelihood trees were computed as unrooted and were rooted *a posteriori* in FigTree ver. 1.2.3 (Andrew Rambaut^9^) with the midpoint method.

The 3D structure of proteins was constructed using the Phyre^2^ server^10^ (Kelley et al., 2015). Profiling of transmembrane domains was done using the TMHMM tool^11^ and functional annotation of NIP2-1 like proteins was performed using the Conserved Domain Database^12^. Amino acids were aligned in CLC Sequence Viewer (version 7.7.1) for visualization of NPA motifs and ar/R selectivity filters in PdNIP2 proteins.

The Statgraphics Centurion (version 15.2.05) and Microsoft Excel 365 software were used for statistical evaluation. The differences among group means were assessed by ANOVA (analysis of variance) and LSD (least significant difference) served as a *post hoc* test. Data from qPCR were evaluated by Student’s *t* test (Microsoft Excel). Statistical significance was attributed at the 0.05 probability level.

## Results

### Silicon Accumulation

**FIGURE 1:**
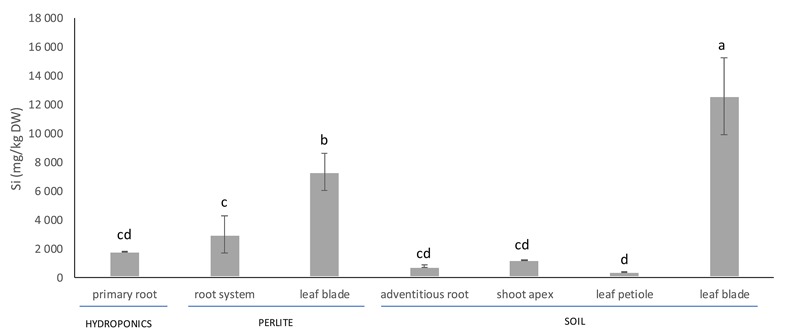
Silicon concentration in vegetative organs of *Phoenix dactylifera* cultivated in hydroponics, perlite or soil. The plants cultivated in perlite were 1-year-old in comparison to the well-developed, 10-year-old plants grown in a soil. Different letters indicate significant differences between the treatments at 0.05 level. Values are means (*n* = 4) ± standard deviation.

Silicon accumulated in all organs of the date palm plants studied (Figure 1). The concentration of Si in plant tissues varied according to the developmental stage of plants and the cultivation method. The largest Si concentration was found in leaf blades of plants, whether cultivated in soil or perlite (Figure 1). The average concentration of Si in leaf blades of 10-year-old palm plants reached ca. 13 g kg^-1^ dry weight (1.3% dry weight). The shoot apexes, leaf petioles and adventitious roots of 10-year-old plants had significantly lower Si concentrations than the leaf blades (Figure 1). The Si concentration in leaf blades of 1-year-old plants grown in perlite was significantly less than that in leaf blades of 10-year-old plants grown in soil, whereas the Si concentration in roots of 1-year-old plants grown in perlite was significantly larger than that of 10-year-old plants grown in soil (Figure 1). The Si concentration in primary roots of plants grown hydroponically was not significantly different from the Si concentration in roots of plants cultivated in perlite or soil.

### Anatomy of Vegetative Organs and Si Deposits

**FIGURE 2:**
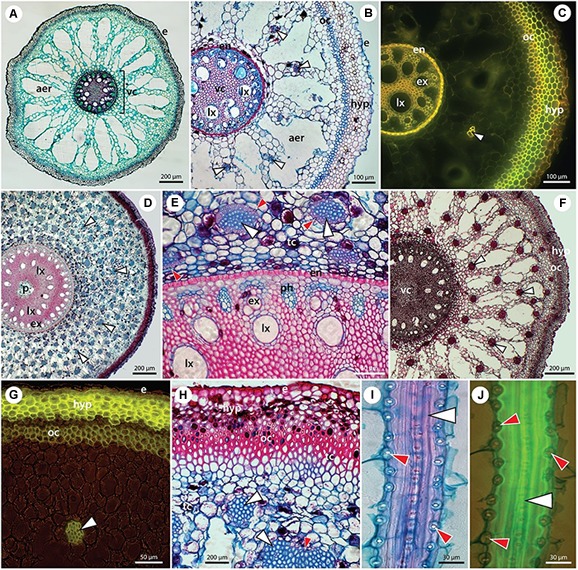
The anatomy of individual root types in *P. dactylifera* with fiber bands and stegmata – specialized Si-accumulating cells. **(A)** Seminal root. **(B,C)** Lateral roots. **(D–J)** Adventitious roots in transverse **(D–H)** and longitudinal sections **(I,J)**. Paraffin embedded sections stained with alcian blue/safranin **(A,B,D,E,H–J)** or with Basic fuchsin **(F)**, unstained – autofluorescence **(C)**, and hand sections stained with Fluorol yellow in UV light **(G)**. **(A)** Seminal root of 2-month-old seedling covered by rhizodermis (epidermis) (e) and composed hypodermis. Mid-cortex typically develops an extensive aerenchyma (aer). Polyarch vascular cylinder (vc) with 11 alternating xylem and phloem poles, several late metaxylem vessels are shifted centripetally and sclerified pith is located in the center. No fiber bands are present at this stage of development. **(B,C)** Lateral roots structurally resemble the seminal root, with exception of fiber bands (white arrowheads) regularly scattered in the cortex. Stegmata cells, adjacent to the fiber bands, are not visible at this magnification. Rhizodermis (e) and composed hypodermis (hyp) are formed by several layers of cells with varying cell wall thicknesses. Extensive aerenchyma (aer) occupies the mid-cortex. Endodermis (en) with thick U-shaped inner tangential walls. Broad late metaxylem vessels (lx) are shifted centripetally from the xylem poles formed by early metaxylem vessels (ex). **(D–J)** Adventitious roots of adult plants are characterised by a multitude of fiber bands (white arrowheads) scattered in the mid-cortex. Proportionally to the age/thickness of the adventitious roots, the number of fiber bands counts from dozens **(F,G)** to hundreds **(D,E,H)**. Composed hypodermis (hyp) is formed by several layers of exodermis with suberized cell walls **(G,H)**. Outer cortex is located internally to the hypodermis, composed of several layers of sclerenchyma. Many cells in the peripheral tissues contain tannins (tc). Polyarch vascular cylinder with several dozens of alternating xylem (ex) and phloem (ph) poles is surrounded by thick-walled endodermis (en). An additional circle of late metaxylem (lx) vessels is present centripetally from xylem poles. In thick roots, the pith (p) might form a central cavity. **(E)** Stegmata with silica phytoliths (red arrowheads) are attached to the surface of fiber bands and are clearly visible in longitudinal sections of roots **(I,J)**.

**FIGURE 3:**
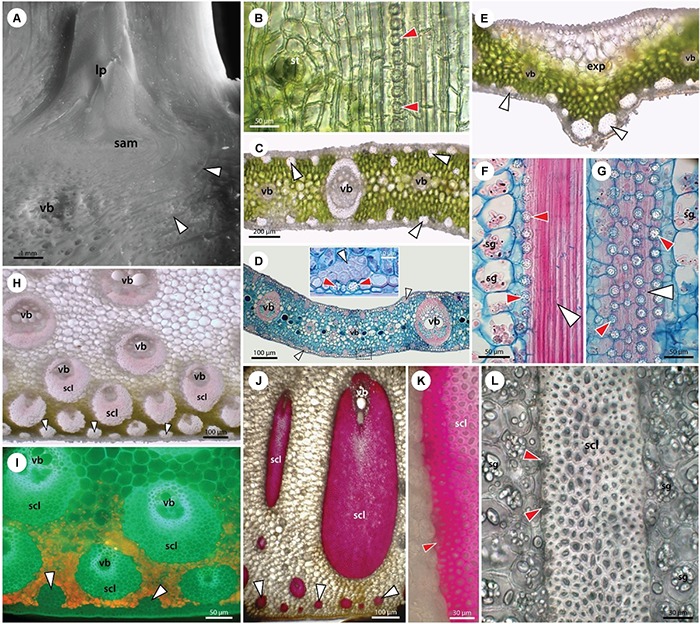
Structure of stem apex **(A)**, young leaf **(B)**, leaflet blade **(C–G)**, leaf petiole **(H,I)**, and leaf sheath **(J–L)**. Stegmata in leaves are associated with both fiber bands and vascular sclerenchyma. **(A)** Stereomicroscope image of the shoot apex showing the shoot apical meristem (sam), leaf primordia (lp), vascular bundles (vb), and fiber bands (white arrowheads). **(B)** Unstained paradermal section of a young leaf from a date palm seedling showing epidermal cells, stoma (st), and subepidermally occurring stegmata (red arrowheads) attached to the surface of a fiber band. **(C–G)** Adult leaflet from a 10-year old. Unstained sections **(C,E)** show epidermal and hypodermal layers at the leaf surface, mesophyll with vascular bundles (vb), and fiber bands (white arrowheads) occurring both adaxially and abaxially. In the central part of the leaflet the mid vein is absent **(E)** and expanding tissue of large parenchyma cells is present adaxially (exp). In the opposite-abaxial part, two large fiber bands are developed. Stegmata adjacent to the fiber bands and sclerenchyma sheaths of vascular bundles can be seen in high magnification **(D)** and more clearly in longitudinal sections **(F,G)**. Alcian blue/safranin stained samples **(D,F,G)**. **(H,I)** Leaf petiole and **(J–L)** leaf sheaths of adult 10-year-old plant in unstained cross-sections are shown either under white light **(H,J,L)** or UV irradiation (I). **(J,K)** Phloroglucinol-HCl staining visualizing cell wall lignification. Leaf petiole and leaf sheath are covered by a single layer epidermis and lignified hypodermis **(I,J)**. Mesophyll is composed of chlorenchyma **(H,I)** and parenchymatous ground tissue. Peripherally present fiber bands (white arrowheads) and large sclerenchyma sheaths of vascular bundles are accompanied by stegmata, as seen in high magnification **(F,G,K,L)**. The ground tissue cells usually contain a number of starch grains (sg).

The structural organization of date palm, with the focus on Si deposition, is summarized in Figure 2, 3 and Supplementary Figure S1. Silicon deposits are present in the form of silica aggregates, termed phytoliths, in specialized small cells, termed stegmata. Stegmata in roots are exclusively attached to the sclerified bundles of fibers in the cortex. These bundles occur rarely in primary seminal roots (Figure 2A) but can be numerous in lateral roots (Figure 2B,C) and adventitious roots (Figure 2D–J). In the thinnest laterals (≤1 mm diameter), only individual bundles formed by 2–4 fibers are developed (Figure 2C). In thicker laterals (≥1 mm) one circle of fiber bundles is present formed by ∼10 fibers (Figure 2B). In the thickest adventitious roots, the number of fiber bundles can exceed 100 and they are scattered within the whole mid cortical region (Figure 2D,F).

Here, we have studied relatively young date palm plants and focus on the presence of sclerifying sheaths of vascular bundles and leaf traces occurring close to the shoot apex (Figure 3A). Already these sheaths are accompanied by stegmata accumulating Si.

The anatomy of the simple leaves of young plants is similar to the leaflets of the compound leaves of adult plants (Figure 3B–E). Stegmata with phytoliths are present in leaves in two anatomically distinct locations. One location is around the isolated bundles of sclerenchyma fibers occurring immediately subepidermally or deep in the mesophyll covered with axially arranged rows of stegmata (Figure 3F,G). The second location is around the sheath of sclerenchyma fibers surrounding the veins with a collateral arrangement of vascular tissues. The stegmata occurring in the petioles and leaf sheaths are of the same type and distribution as in the leaves and leaflets (3 H–L).

### SEM/EDX and Raman Analysis of Si Phytoliths

**FIGURE 4:**
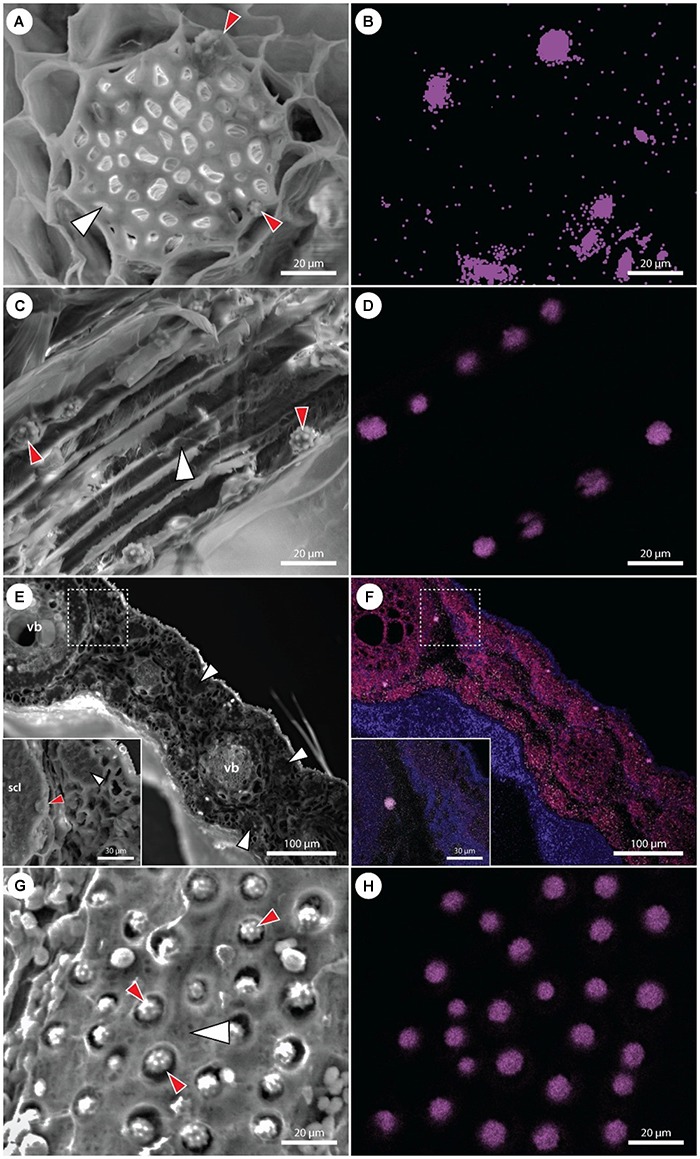
Scanning electron microscopy images of various *P. dactylifera* tissues with corresponding maps showing the distribution of Si (violet color). **(A,B)** Cross section of an adventitious root showing detail of a fiber band (white arrowhead) with adjacent stegmata cells containing Si phytoliths (red arrowheads). Multiple phytoliths are not visible in **(A)**, though detected by EDX **(B)**. **(C,D)** Longitudinal section through the fiber band (white arrowhead) in an adventitious root. Cell walls of several stegmata cells are disrupted, uncovering Si phytoliths (red arrowheads). **(E,F)** Cross section of a leaf showing the presence of Si phytoliths in stegamata cells associated with vascular bundles (vb) and fiber bands (white arrowheads). A detail on a stegma (red arrowhead) associated with the vascular bundle sclerenchyma (scl). **(G,H)** A surface view on a fiber band (white arrowhead) with a dense net of adjacent stegmata. Cell walls of multiple stegmata are disrupted, uncovering Si phytoliths (red arrowheads).

A detailed investigation of various date palm tissues was performed to detect the pattern of Si distribution using SEM coupled with X-ray analysis of element distribution (EDX). In roots, stegmata cells containing Si aggregates were positioned on the outer surface of the sclerenchyma bundles (Figure 4A,B), organized in rows of cells with an average distance between the individual phytoliths of about 10–12 μm and an average size of Si phytoliths of between 6 and 8 μm (Figure 4C,D). Silicon is also present in the shoot apex, mostly in the form of individual Si phytoliths associated with the sclerenchyma of the vascular bundle. In leaves, the X-ray analysis showed that Si was localized in leaf tissues at two sites: as a part of sclerenchyma around the vascular bundles, and as a part of individual sclerenchyma bundles in the leaf mesophyll. Silicon was not detected in the epidermis, nor in association with the cuticle (Figure 4E,F). A very dense net of Si aggregates was observed in the leaf sheaths. The size of Si phytoliths varied between 5 and 10 μm, and they were associated with the surface cell layers of sclerenchyma bundles (Figure 4G,H).

**FIGURE 5:**
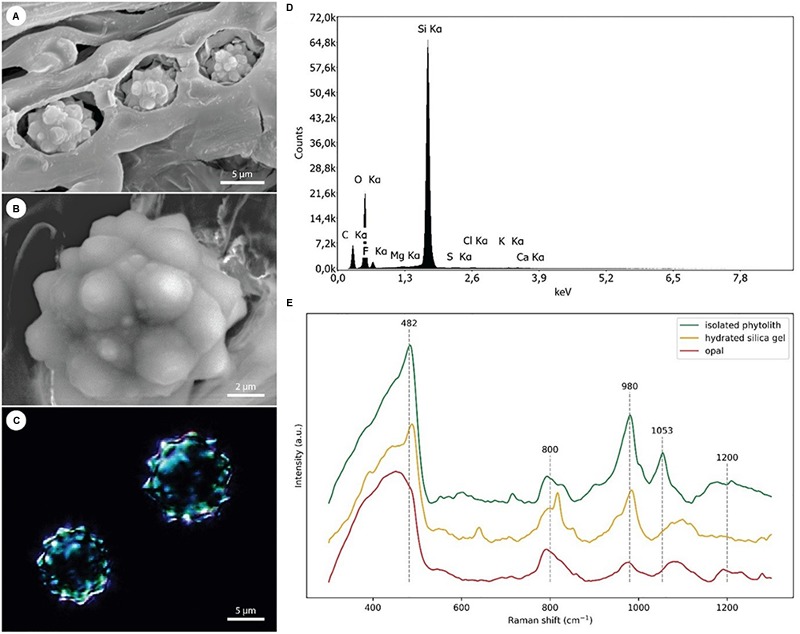
EDX and Raman analyses of the Si phytoliths. **(A)** Scanning electron micrograph showing three adjacent stegmata with disrupted cell walls exposing the Si phytoliths. **(B)** Detail of an exposed phytolith used in EDX analysis. **(C)** Dark-field microscopy image of isolated Si phytoliths used in Raman analysis. **(D)** A representative spectrum from EDX analysis of a Si phytolith demonstrating the dominance of Si and O as its main chemical constituents. **(E)** Comparison of Raman spectra collected from isolated silica phytoliths [shown in panel **(C)**], hydrated silica gel, and opal.

In general, stegmata were almost entirely filled by Si phytoliths (Figure 5A–C). X-ray analysis of the surface elemental composition of phytoliths revealed two major elements, Si and oxygen (Figure 5D). The presence of carbon was attributed to the surface carbon coating of samples prior the analysis. No other elements were detected in phytoliths.

Representative Raman spectra of isolated silica phytoliths and reference spectra of hydrated silica gel and opal were compared (Figure 5E). All three spectra were dominated by a broad band in the region 400–490 cm^-1^ assigned to Si–O–Si bond-rocking vibration, underlining the amorphous nature of the silicas. In contrast to spectra from opal, spectra from both phytoliths and silica gel exhibited a well-resolved peak near 482 cm^-1^. The broad and asymmetrical band around 800 cm^-1^ visible in all three spectra was assigned to symmetric Si–O–Si stretching vibrations arising from the heterogeneities in the geometry of SiO_2_ subunits. The Si–O vibrations of non-bridging oxygen within the region 950–1000 cm^-1^ reflects the abundance of Si–OH groups (985 cm^-1^) and the presence of chemical impurities. Whereas the opal spectrum showed relatively low abundance of Si–OH groups, illustrating its compact inner structure, both phytoliths and silica gel exhibited a relatively high abundance of Si–OH groups, indicating a large surface area. A band assigned to asymmetric Si–O–Si stretching vibrations is located between 1050 and 1200 cm^-1^. Here, the phytolith spectra exhibit a peak around 1053 cm^-1^, indicating that some other elements or contaminants might be present.

### Raman Analyses of the Cell Wall Composition

Raman microspectroscopy was used to investigate the cell wall composition of root and leaf tissues (Supplementary Figure S2). In roots, the relatively thin hypodermal cell walls exhibited signals indicative of suberization and intense lignification with balanced S/G-lignin ratio, relatively high H-lignin content and ferulic/p-coumaric acids. The outer cortical layer displayed similar cell wall composition to the cortical fiber bundles, characterized by high cellulose crystallinity, relatively weak lignification of the cell wall, but intense lignification in the compound middle lamellae. The thin-walled cell strands separating the aerenchyma lacunae in the mid cortex showed high abundance of both aromatic and aliphatic esters. The cell walls of the inner cortex displayed a low abundance of phenolic compounds (1600–1660 cm^-1^), but their ester-rich constitution was indicated by a broad band between 1660 and 1750 cm^-1^ (C=C and C=O stretching). These cells probably represented an early developmental stage of the thin-walled cells of the mid cortex. The endodermis has developed a thick U-shaped cell wall with relatively high content of phenolic compounds (including H-lignin) in comparison to the thin-walled cells of the mid cortex as well as to the fiber walls. In addition, multiple signals associated with lipidic substances indicated suberin deposits and a relatively large amount of ferulic/p-coumaric acid.

The cell walls of the pith sclerenchyma exhibited a qualitatively similar composition to the outer cortex but with a slightly higher degree of wall lignification. Early metaxylem walls were heavily lignified with a high S/G-lignin ratio. The spectra from late metaxylem walls exhibited a very similar profile, but with less wall lignification. The phloem cell walls exhibited a profile associated with simple primary cell walls, displaying a relatively high pectin signal (817 cm^-1^), a very low signal from phenolic compounds, low cellulose crystallinity and a relatively high abundance of hemicelluloses (region 470–515 cm^-1^, 1462 cm^-1^).

### Phylogenetic Placement of Si Transporters From Date Palm

**FIGURE 6:**
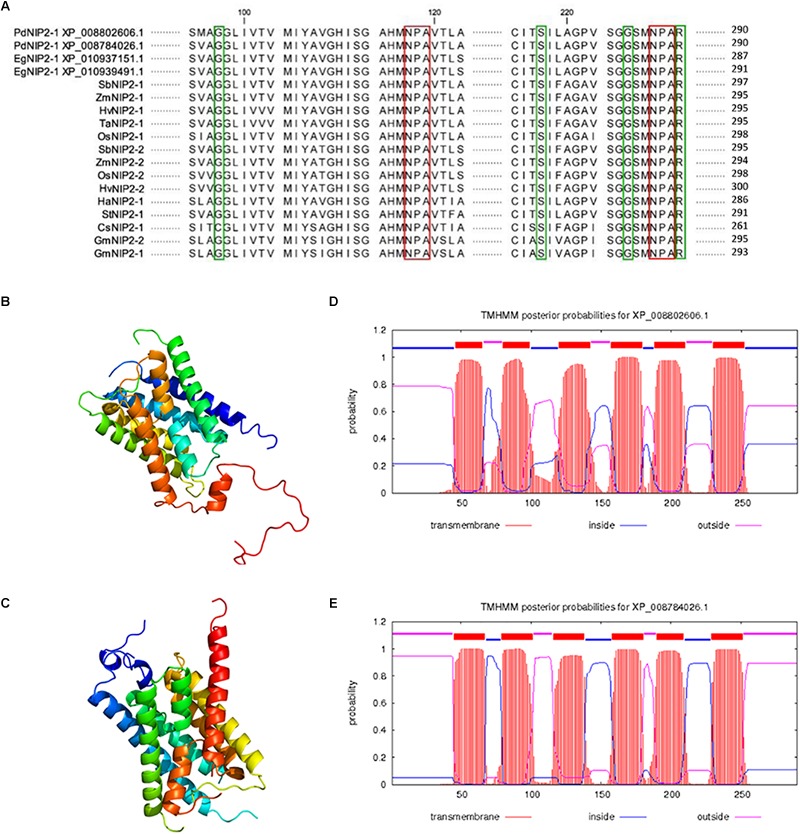
Alignment of amino acid sequences of silicon influx transporters (NIP2s) in various plant species with highlighted NPA motifs (red color) and the G-S-G-R Ar/R selectivity filter (green color) **(A)**. Prediction of the 3D structure of PdNIP2-1 **(B)** and PdNIP2-2 **(C)** proteins. Prediction of transmembrane domains of PdNIP2-1 **(D)** and PdNIP2-2 **(E)** proteins.

Two putative Si transporters, PdNIP2-1 (XP 008802606.1) and PdNIP2-1 (XP_008784026.1) share an 87% identity based on a BLAST alignment and both show the hallmark features required for Si transport (Figure 6A). The 3D model of both proteins showed an hourglass-like structure (Figure 6B,C). The TMHMM tool for prediction of transmembrane domains showed six transmembrane helices for both proteins, identical to the known Si transporters of other plant species (Figure 6D,E). Both proteins were classified functionally as membrane channels that are members of the MIP superfamily using this tool.

**FIGURE 7:**
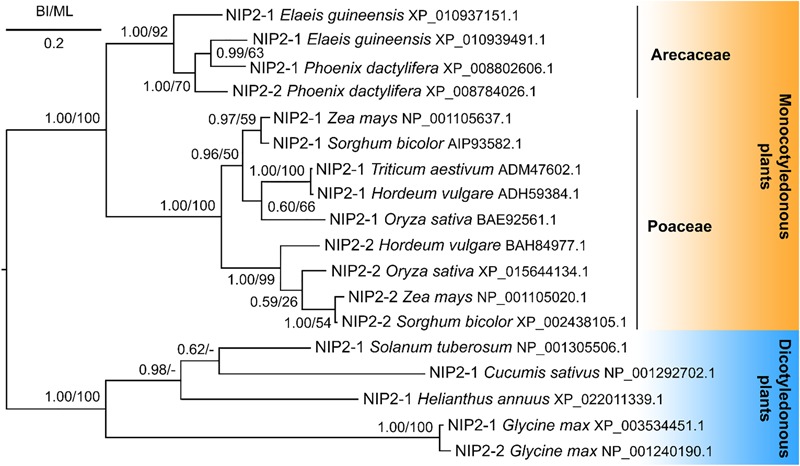
Phylogeny of silicon influx transporters (NIP2s) of monocotyledonous and dicotyledonous plants. Posterior probabilities for Bayesian inference and bootstrap values for maximum likelihood were mapped onto the 50%-majority rule consensus tree. The scale bar indicates two substitutions per ten hundred amino acid positions.

Phylogenetic analyses showed that the PdNIP2-1 and PdNIP2-2 transporters from date palm belong to the well-defined group of Si influx transporters previously identified in various plant species (Figure 7). Transporter sequences from Arecaceae were clustered together with strong statistical support in Bayesian and maximum likelihood trees. In both phylogenetic analyses, sequences from the family Poaceae were classified in a sister position to those from the Arecaceae, supporting a common phylogenetic ancestry of Si transporters in the monocotyledonous cluster. The NIP2 transporters from dicotyledons formed a distinct, statistically fully supported group (Figure 7).

### Silicon Permeability of PdNIP2-1

**FIGURE 8:**
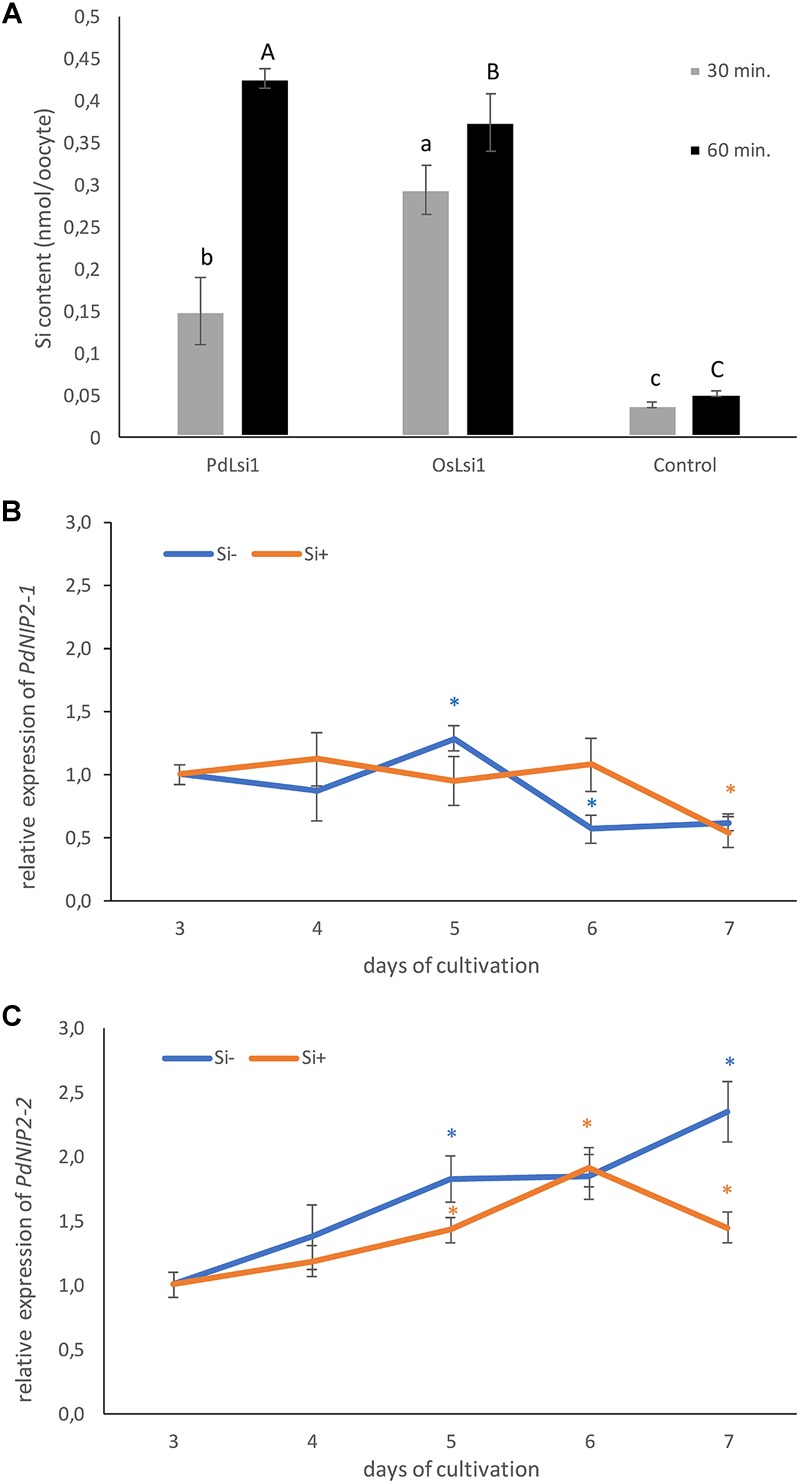
**(A)** Silicon influx transport activity of PdNIP2-1 from date palm evaluated at two different time points in *Xenopus oocyte* assays. Oocytes injected with OsLsi1 from rice, or water were used as positive and negative controls, respectively. Values are means ± standard deviation. Different letters indicate significant differences in the same time point. The relative transcript level of *PdNIP2-1* **(B)** and *PdNIP2-2* **(C)** genes in roots of hydroponically grown date palm seedlings in the Si– treatment (orange line) and the Si+ treatment (blue line) from the third to the seventh day of cultivation. Gene expression for the control was set as 1.0. Statistically significant differences between control and treated plants were analyzed by Student’s *t* test and are denoted as ^∗^*P* < 0.05. Values are means ± standard deviation. The mean values are based on three technical and three biological replicates.

To prove the functionality of *PdNIP2, X. laevis* oocytes expressing *PdNIP2-1* were assayed for their ability to accumulate Si (Figure 8A). Oocytes expressing either *PdNIP2-1* or rice *OsLsi1* accumulated significantly more Si than oocytes injected with water, and the same amount after 60-min incubation, confirming the function of *PdNIP2-1* as a Si transporter as predicted from *in silico* analyses (Figure 8A).

### Expression of PdNIP2 Si Transporters in Roots of Date Palm Plants

The expression of *PdNIP2* genes in roots of date palm plants was constant. The relative amount of the *PdNIP2-1* transcripts in roots showed only slight daily variation (Figure 8B), varying between 0.62–1.29 and 0.54–1.12 for mRNA in the Si- and Si+ treatments, respectively. The second transcript *PdNIP2-2* showed a general increase in expression with length of cultivation in both Si- and Si+ treatments (Figure 8C). However, the fold change of this transcript ranged only between 1.0 and 2.35 in the Si- treatment and 1.0–1.92 in Si+ treatment. Because this variation of both transcripts is rather low, we also used the BestKeeper tool to determine the stability of expression of the transcripts in the Si- and Si+ conditions, based on the correlation coefficient of all possible pairs of the candidate reference genes (Supplementary Table S4). Both transcripts showed a low (<1) standard deviation of the threshold cycle values (SD C_T_) and a low SD (<2) of the fold change of gene expression (x-fold), with a strong correlation for all transcripts (Supplementary Table S4).

## Discussion

There is little knowledge of the role of Si in date palm, with limited data being available (Fathi, 2014). The present study might stimulate research on this important element in this economically (FAOSTAT, 2019) and medicinally (Zhang et al., 2017) important species. This study provides conclusive evidence of the presence and functionality of Si influx transporters in date palm and highlights a unique pattern of Si deposition in stegmata cells. Stegmata containing Si phytoliths are present in all organs of the date palm, attached to the surface of sclerenchyma bundles in roots, leaves and stem and to the surface of sclerenchyma sheaths of vascular bundles in stems and leaves (Figure 2–4).

### Morphology of Phytoliths

The phytoliths of *Phoenix dactylifera* are classified as spherical, with surface appearance ranging between warty and echinate/spiculate (Figure 5) (Prychid et al., 2003; Tomlinson et al., 2011). Such morphology is recognized as typical for palm species and provides a reliable taxonomical identifier (Piperno, 2006; Tomlinson et al., 2011). The hat-shaped/conical phytoliths are the only other morphotype found in palms and can be found, for example, in *Caryota, Sclerosperma*, and *Reinhardtia* (Tomlinson et al., 2011). In contrast, grass phytoliths seem to exhibit much greater morphological variability, where it is possible to identify several morphotypes within the leaf epidermis alone (Kumar et al., 2017).

Mature stegmata possess thick inner tangential and radial cell walls and thin primary outer tangential walls (Figure 2, 3). In the majority of cases, each stegmata contains a single phytolith that occupies almost the entire cell volume. The size of stegmata varies between 10 and 12 μm and the size of phytoliths varies between 6 and 8 μm.

### Phytolith Structure

Raman microspectroscopy confirmed the amorphous nature of the silica framework (a broad band in region 400–490 cm^-1^), which is a well-known attribute of silica phytoliths in general (Currie and Perry, 2007). A well-resolved peak near 482 cm^-1^ and a relatively strong signal near 985 cm^-1^ further indicated a large surface area of the silica and suggested that the phytoliths have a microporous structure (Iqbal and Vepřek, 1982; Gailliez-Degremont et al., 1997). This is consistent with the study by Lins et al. (2002), revealing the porous structure of phytoliths in the palm *Syagrus coronata*. A high abundance of superficial –OH groups might favor the adsorption of new silica species via hydrogen bonding (Coradin and Lopez, 2003) and enable the growth of the phytolith. According to Lins et al. (2002) the phytoliths of *S. coronata* were composed of granules of varying size and morphology. This feature is reflected in the Raman spectra by a broad band around 1200 cm^-1^, indicating that multiple degrees of silicate unit polymerization are present in the phytoliths of date palm (McMillan and Remmele, 1986). This might have resulted from contaminants disrupting the silica framework during polymerization (McMillan, 1984; Marsich et al., 2009).

### Phytolith Association With Cell Walls

The phytoliths from date palm do not seem to contain any organic backbone (Figure 5), which was also reported for the palm *S. coronata* (Lins et al., 2002). In contrast to palms, the phytoliths of grasses are typically associated with the cell walls, particularly, within lignified tissues (Guerriero et al., 2016; Kumar et al., 2017). Raman signals from their scaffolding organic materials can be detected even if harsh procedures are used to isolate phytoliths (Gallagher et al., 2015), usually indicating the presence of phenolic compounds and hemicelluloses (Guerriero et al., 2016; Soukup et al., 2017). Recent studies suggest that lignification might be required to initiate silica deposition (Zhang et al., 2013; Soukup et al., 2017). The association of phytoliths with lignified cell walls has also been reported in dicots, despite the fact that they have low tissue Si concentrations (Scurfield et al., 1974; Hodson et al., 2005). It is speculated that a trade-off between the accumulation of silica and lignin might occur in plants (Schoelynck et al., 2010; Yamamoto et al., 2012; Klotzbücher et al., 2018). Such a phenomenon is often considered beneficial, with the cost of silicification being estimated to be only 3.7% that of lignification (Raven, 1983). However, although the stiffness provided by these two components might be comparable, they are not entirely interchangeable due to the much lower density of lignin and its water repelling properties (Raven, 1983; Soukup et al., 2017). However, unlike grasses, Si phytoliths in palms are probably formed intracellularly in the vacuole and seemingly without an organic backbone. Schmitt et al. (1995) performed a detailed TEM study of stegmata ontogenesis in the rattan palm species *Calamus axillaris*. They concluded that the “silica-body” grows within the vacuole. The growth of the “silica body” is probably controlled via active Si accumulation progressively supersaturating the vacuole, and by additional modulation of its physico-chemical environment.

Anatomical observations of date palm show that stegmata that are almost completely filled with Si phytoliths are very abundant near lignified tissues, principally in the outer surface of the sclerenchyma bundles (fibers) in roots, stem and shoots of date palm (Figure 2, 3). Therefore, we also performed Raman spectra analysis of cell walls in lignified tissues. Despite Raman spectra from the fiber cell walls indicating relatively weak lignification, they exhibited good responsiveness to Wiesner reaction (phloroglucinol-HCl). This can be associated with a relatively high abundance of sinapyl/coniferyl aldehydes, which are the key cell wall reagents in this reaction. Furthermore, high content of phenolic aldehydes in the lignin polymer indicate an early stage of lignification (Pomar et al., 2002). In older tissues, additional H-lignin signals appeared in the spectra and the S/G-lignin ratios declined, suggesting that in later stages of the cell wall development predominantly G- and H-lignin were deposited. Relatively weak lignification of the cell wall, high cellulose crystallinity and strong lignification of the compound middle lamellae indicate the gelatinous character of these fibers (Mellerowicz and Gorshkova, 2012). As such, these fibers might provide adjustable mechanical support, that is gradually stabilized by the deposition of lignin as the tissue matures and the organ achieves its optimal position in the environment. This anatomical trait might have substituted for secondary growth, allowing palms to achieve a stable erect posture of the trunk with much lower metabolic costs invested into rigid mechanical tissues.

### The Role of Silica Phytoliths

Silica phytoliths are traditionally perceived as structures supporting the mechanical properties of plant tissues (Currie and Perry, 2007; Yamanaka et al., 2009). The abrasive nature of silica also deters grazing animals and phytophagous insects (Massey and Hartley, 2009). Moreover, leaf phytoliths might facilitate the transmittance of light to the mesophyll and improve the efficiency of photosynthesis (Sato et al., 2016). Despite these benefits, demands driving the evolution of silica phytolith formation are still unclear (Strömberg et al., 2016). A contrasting evolutionary perspective views silicic acid as a potentially toxic substance and controlled silicification as a mechanism for its detoxification (Exley, 2015). In concentrations exceeding 2 mM, silicic acid is prone to polymerize and might lead to silica scaling on the surfaces of membranes or enzymes and impair their functionality. On the other hand, it offers protection against fungi and insects and might stabilize the membrane against harmful effects (Coskun et al., 2018). So far, the roles of silica phytoliths in palms have not been assessed experimentally. Besides possible prophylactic roles, intracellular formation of Si phytoliths might indicate a role in harnessing excess Si accumulated by the plant. This might be crucial for the longevity of palm tissues and/or slow progression of fiber lignification. For instance, up to 20–30% less lignin was recorded in rice straws due to the silica-lignin trade-off (Klotzbücher et al., 2018), and in aged bamboo leaves, epidermal silicification was reported to extend to chlorenchyma and reduce the leaf photosynthetic efficiency (Motomura et al., 2008). Curiously, a negative correlation between leaf longevity and silicon content was found across various plant groups (Cooke and Leishmann, 2011).

### Molecular Aspects of Si Transport

The Lsi1 transporter, which mediates Si influx to roots, was first discovered in rice plants (Ma et al., 2006). Since then, the list of plant Si transporters has been extended to include those of many other species (Yamaji et al., 2008; Chiba et al., 2009; Mitani et al., 2009, Mitani-Ueno et al., 2011; Montpetit et al., 2012; Vivancos et al., 2016; Ouellette et al., 2017). The NIP2 transporters, especially the well characterized NIP2-1 (Lsi1), have a role in Si uptake from soil to root cells and are, therefore, intimately involved in Si accumulation by flowering plants. In addition, a more efficient NP3,1 aquaporin has been identified in horsetail (*Equisetum arvense*) that contains a STAR pore in contrast to the GSGR pore in monocots including date palm (Grégoire et al., 2012). However, in our study, we take only NIP2 proteins into consideration. For this reason, we focused the molecular study on the properties of PdNIP2 in date palm roots. In our study, transcriptomic data, bioinformatic analyses and oocyte assays revealed the presence and functionality of PdNIP2 transporters in *P. dactylifera* similar to those known in other plants. These proteins share the hallmark features, such as the ar/R pore and the 108 amino acid sequence between the NPA loops required for Si transport across the plasma membrane (Deshmukh et al., 2015). The permeability of *PdNIP2-1* to Si was proven to be comparable to that of rice Lsi1 using a *Xenopus* oocyte bioassay, a heterologous expression system that has proven reliable for testing the functionality of Si transporters. Using Si as a substrate rather than germanium, our data have also eliminated any possible complication associated with a surrogate substrate (Garneau et al., 2018). A phylogenetic analysis clustered the sequences of NIP2 transporters from the Arecaceae separately, but in a sister position to the Poaceae.

The expression of *PdNIP2* genes in roots of hydroponically grown date palm plants was relatively unaffected by the presence or absence of Si in the growth medium (Figure 8). Plant species appear to differ in the effects of rhizosphere Si supply on the expression of NIP2 genes and their expression can be up-regulated, down-regulated or unaffected by Si addition to cultivation media (for a detailed review, see Ma and Yamaji, 2015). Analysis using the BestKeeper tool suggests that both *PdNIP2-1* and *PdNIP2-2* have the transcriptional attributes of a reference gene, although *PdNIP2-1* is a better reference gene than *PdNIP2-2*. It is possible that the constitutively large Si accumulation in *P. dactylifera* plants might be a consequence of the relatively high stable expression of the *PdNIP2* genes that are most probably responsible for Si uptake. In contrast, plants that do not accumulate Si, especially dicots, have a constitutively low expression of NIP2 genes that is even supressed by the presence of Si in cultivation media, as for example NIP2-1 (XM_013836541) in *Brassica napus* (Haddad et al., 2019), which might explain smaller accumulation of Si by dicots than monocots. We found homologous sequences to *OsLsi2* and *OsLsi6* transcripts in the sequence of *P. dactylifera*. It is, therefore, suggested that the uptake of Si from soil into root epidermal cells is mediated by PdNIP2. Silicon is subsequently transported from cortical cells to the xylem by a Lsi2-like protein and translocated and distributed in leaves by a Lsi6-like proteins.

## Conclusion

In conclusion, Si is accumulated in all tissues of *P. dactylifera* plants, where Si aggregates are present in stegmata. In contrast to grasses, in which Si is generally associated with epidermal tissues, the stegmata of palms are abundant in the outer surface of the sclerenchyma bundles (fibers) present in roots, shoot apex, leaf petioles and blades with the diameter of Si aggregates/phytoliths ranging from 6 to 8 μm. The surface of phytoliths is composed of only silicon and oxygen, without any organic constituents. The analysis of the fiber cell walls suggests they possess a gelatinous character and together with Si phytoliths might provide strong mechanical support for the plant. Again, in contrast to grasses, in which Si phytoliths are mostly associated with cell walls, those of *P. dactylifera* appear to be formed intracellularly. As *P. dactylifera* is a Si accumulator homologous sequences of *Lsi* genes typical for grasses, which are also Si accumulators, were predicted from its genome and found to be functional. Phylogenetic analysis of those transporters within Arecaceae, suggested that they occupied a sister clade to those of the Poaceae and both were distinct from those of dicots. It is likely that, as the palms and grasses diverged, different patterns of Si accumulation became established in each clade.

## Data Availability

All datasets generated for this study are included in the manuscript and/or the Supplementary Files.

## Author Contributions

BB, KŠ, and SV carried out the gene expression study, bioinformatic analysis and other molecular biology experiments. PV carried out the phylogenetic analyses. RB, RD, and HS carried out oocyte assays and functional annotation of *NIP2-1* gene. MS carried out the cell wall analysis. AL, MV, MW, and IL carried out the scanning electron microscopy coupled with X-ray microanalysis. AL carried out the anatomical study, designed the research together with PW and HE-S, and supervised the project. All authors discussed the results and commented on the manuscript.

## Conflict of Interest Statement

The authors declare that the research was conducted in the absence of any commercial or financial relationships that could be construed as a potential conflict of interest.

## Footnotes

1 http://www.effemm2.de/spectragryph/

2 http://www.qiagen.com/

3 http://www.roche.com

4 http://www.invitrogen.com/site/us/en/home/brands/ambion.html

5 http://primer3plus.com/web_3.0.0/primer3web_input.htm

6 http://guidance.tau.ac.il/ver2/

7 http://www.phylo.org

8 http://www.atgc-montpellier.fr/phyml/

9 http://tree.bio.ed.ac.uk/software/figtree/

10 http://www.sbg.bio.ic.ac.uk/phyre2/html/page.cgi?id=index

11 www.cbs.dtu.dk/services/TMHMM/

12 www.ncbi.nlm.nih.gov/Structure/cdd/cdd.shtml
