# Supplementary material for: Silicon Uptake and Localisation in Date Palm (Phoenix dactylifera) – A Unique Association With Sclerenchyma
Source: Front Plant Sci. 2019 Aug 13;10:988. doi: 10.3389/fpls.2019.00988 (PMC6701203; doi:10.3389/fpls.2019.00988)
Supplement: Supplementary file 1 [file Table_1.DOCX]

**SUPPLEMENTARY FIGURES AND TABLES**


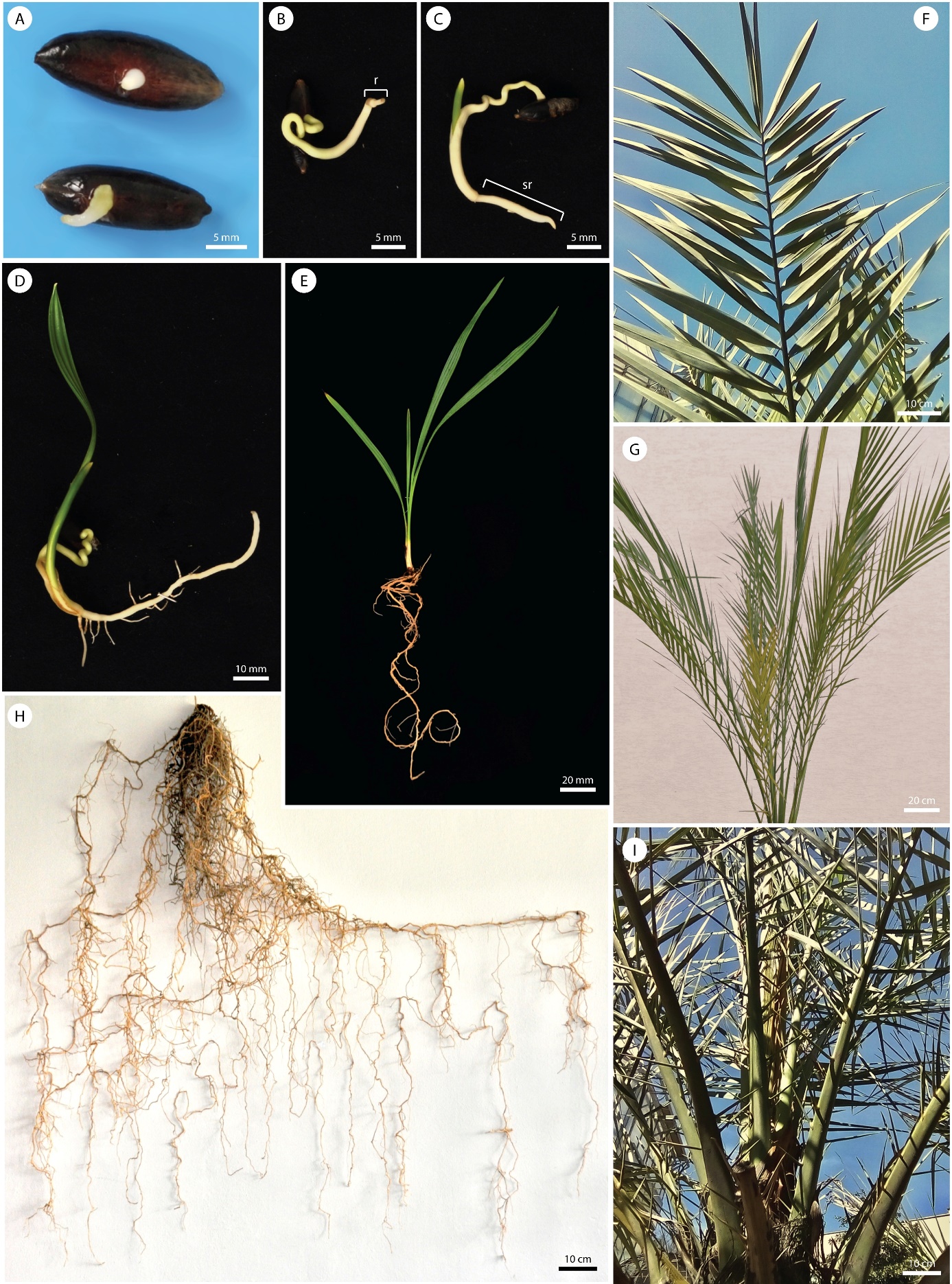


**Figure Supplementary 1.** *Phoenix dactylifera* development and organ morphology.

**A/** Early stage of germination.

**B/** A seedling with long and twisted sheathing portion of a cotyledon and a short radicle (r).

**C/** Seminal root (sr) starts to grow in coordination with the first foliage leaf which emerges from a slit-shaped opening in the sheathing portion of the cotyledon.

**D/** A two-month-old seedling with first foliage leaf and seminal root forming multiple laterals.

**E/** A one-year-old seedling with developing fourth foliage leaf and several adventitious roots in addition to the seminal root. Unlike adult plants, young plants develop only simple leaves.

**F, G, I/** Compound leaves of 10-year-old plants have a typical pinnate arrangement. A massive and long-lasting sheath is attached to the stem, forming its protective covering. A petiole of the leaf axis is extended to the rachis and bears individual leaflets. The basal leaflets are short, thin and spiny, the rest of the leaflets have adaxial V-shape folding.

**H/** The root system of a 10-year-old plant grown in a pot, formed by numerous adventitious and lateral roots.

**
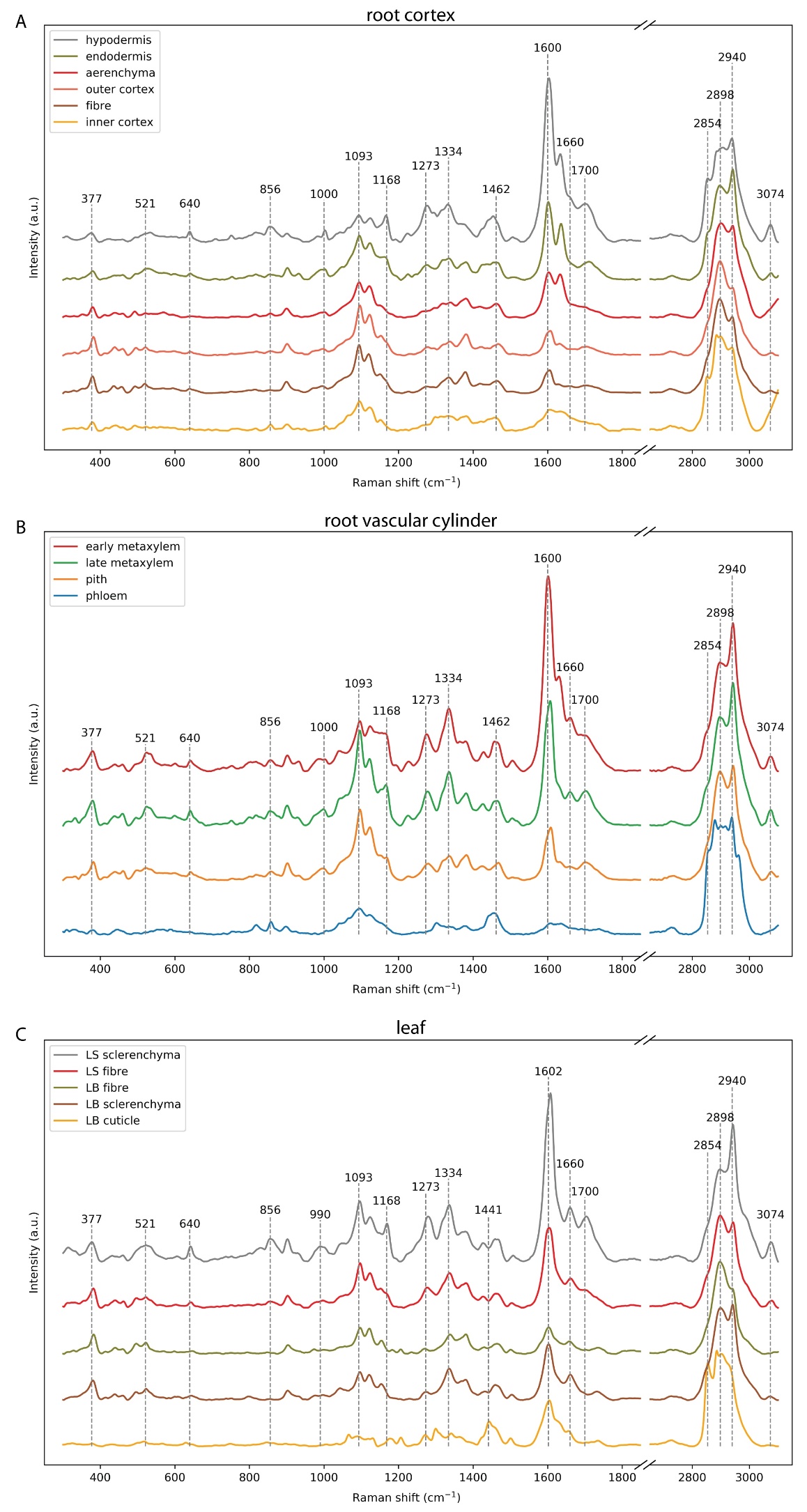
Figure Supplementary 2.** Raman spectra of cell walls obtained from individual tissues in adventitious roots (**A, B**) and leaves (**C**), collected from a 10-year-old plant. Individual baselines were offset to improve the readability of each spectra.

**Table Supplementary 1.** Raman peak assignments for silicate minerals.

| **peak position**  **(cm-^1^)** | **assignment** | **references** |
| --- | --- | --- |
| 400-490 | Si–O–Si rocking | 4 |
| 800 | Si–O–Si stretching (symmetric) | 5, 6 |
| 950-1000 | Si–O (non-bridging oxygen) | 2, 3 |
| 985 | Si–OH | 1, 7 |
| 1050-1200 | Si–O–Si stretching (asymmetric) | 6, 7 |
| [1] Aguiar et al. (2009), [2] Gailliez-Degremont et al. (1997), [3] González et al. (2003), [4] Iqbal and Vepřek (1982), [5] Marsich et al. (2009),  [6] McMillan (1984); [7] McMillan and Remmele Jr. (1986). | | |

**References:**

**Aguiar H, Serra J, González P, León B. 2009.** Structural study of sol – gel silicate glasses by IR and Raman spectroscopies. *Journal of Non-Crystalline Solids* **355**: 475–480.

**Gailliez-Degremont E, Bacquet M, Laureyns J, Morcellet M. 1997.** Polyamines adsorbed onto silica gel: a Raman microprobe analysis. *Journal of Applied Polymer Science* **65**: 871–882.

**González P, Serra J, Liste S, Chiussi S, León B, Pérez-Amor M. 2003.** Raman spectroscopic study of bioactive silica based glasses. *Journal of Non-Crystalline Solids* **320**: 92–99.

**Iqbal Z, Vepřek S. 1982.** Raman scattering from hydrogenated microcrystalline and amorphous silicon. *Journal of Physics C: Solid State Physics* **15**: 377–392.

**Marsich L, Moimas L, Sergo V, Schmid C. 2009.** Raman spectroscopic study of bioactive silica-based glasses: The role of the alkali/alkali earth ratio on the Non-Bridging Oxygen/Bridging Oxygen (NBO/BO) ratio. *Spectroscopy* **23**: 227–232.

**McMillan P. 1984.** Structural studies of silicate glasses and melts-applications and limitations of Raman spectroscopy. *American Mineralogist* **69**: 622–644.

**McMillan PF, Remmele Jr. RL. 1986.** Hydroxyl sites in SiO_2_ glass: A note on infrared and Raman spectra. *American Mineralogist* **71**: 772–778.

**Table Supplementary 2.** Raman peak assignments used in the histology section.

| **peak position (cm^-1^)** | **assignment** | **group** | **component** | **references** |
| --- | --- | --- | --- | --- |
| 377 | C–C–C ring deformation,  C–C–O stretching | polysaccharides | cellulose (crystalline) | 1, 3, 4, 5 |
| 470-515 | coupled modes of heavy atoms,  C–C and C–O stretching | polysaccharides | hemicelluloses | 5, 11, 19 |
| 521 | skeletal deformation | polysaccharides | cellulose | 1, 5, 4, 5 |
| 520-530 | skeletal deformation | phenolic compounds | lignin | 1, 3 |
| 640 | ring and skeletal in-plane deformation | phenolic compounds | lignin (H) | 6 |
| 817 | ring out-of-plane C–OH deformation | polysaccharides | pectin | 16 |
| 856 | C–O–C skeletal mode of α-anomers | polysaccharides | pectin, hemicelluloses | 4, 16, 19 |
| 900 | C–O–C skeletal mode | polysaccharides |  | 14 |
| 980 | C–O stretch and ring mode region | polysaccharides | hemicelluloses | 1, 3, 5 |
| 1040 | C–C, C–O of aryl–O–CH_3_ and aryl–OH stretching | lipids |  | 12, 17 |
| 1060 | alkyl C–C trans and gauche stretches | lipids |  | 12, 19 |
| 1093 | C–O–C glycosidic symmetric stretching | polysaccharides |  | 2, 5, 11 |
| 1123 | C–O–C glycosidic asymmetric stretching | polysaccharides |  | 2, 5, 11 |
| 1141 | aryl–OCH_3_ vibrations,  in-plane aromatic C–H bending | phenolic compounds | coniferyl aldehyde,  sinapyl aldehyde,  lignin (G, S) | 8, 12 |
| 1168 | ring plane deformation C–H,  C–OH stretching | phenolic compounds | ferulic acid,  p-coumaric acid | 5, 9, 10, 13 |
| 1200 | aryl–OCH_3_ | phenolic compounds | lignin (G, S) | 9 |
| 1213 | ring deformation, aryl–OCH_3_ and aryl–OH in-plane bending | phenolic compounds | lignin (H) | 15 |
| 1270 | ring deformation and C═O stretch | phenolic compounds | lignin (G), ferulic acid | 8, 15 |
| 1273 | ═C–H in-plane deformation | lipids | unsaturated fatty acids | 13 |
| 1300 | CH_2_ in-plane twist | lipids |  | 14, 17, 19 |
| 1334 | symmetric C–O–C stretches of two O–CH_3_ groups | phenolic compounds | lignin (S) | 8, 15 |
| 1380 | CH_2_ in-plane bending | polysaccharides | cellulose | 1 |
| 1426 | aromatic skeletal vibrations, CH_3_ bending, aryl–OCH_3_ | phenolic compounds | lignin | 9, 11, 13 |
| 1441 | alkyl CH_2_ bend | lipids |  | 14, 17, 19 |
| 1456 | CH_2_ and C–OH in-plane bending,  CH/CH_2_ wagging | polysaccharides | hemicelluloses | 1, 3, 4, 5, 11 |
| 1600 | aromatic ring C–C stretch | phenolic compounds |  | 6, 8, 9, 10, 17 |
| 1630 | C═C stretching of aromatic sidechain | phenolic compounds | ferulic acid, p-coumaric acid, coniferyl aldehyde, sinapyl aldehyde,  lignin (G, S) | 5, 8 |
| 1650-1660 | C═C stretching | phenolic compounds, lipids | coniferyl alcohol,  sinapyl alcohol, lignin (G,S) | 8 |
| 1700-1750 | ester C═O stretching | lipids, pectins |  | 14, 19 |
| 2854 | CH_2_ symmetric stretching |  |  | 7 |
| 2885 | CH_3_ symmetric stretching |  |  | 14 |
| 2898 | CH_2_ symmetric stretching |  |  | 7, 18 |
| 2940 | CH_2_ asymmetric stretching |  |  | 14 |
| 3008 | ═C–H asymmetric stretching | lipids |  | 14 |
| 3074 | CH stretching |  |  | 6 |
| [1] Agarwal and Ralph (1997), [2] Agarwal et al. (2011), [3] Agarwal (2014), [4] Gierlinger et al. (2008), [5] Himmelsbach and Akin (1998),  [6] Larsen and Barsberg (2010), [7] Littlejohn et al. (2015), [8] Lupoi and Smith (2012), [9] Lupoi et al. (2015), [10] Ma et al. (2014),  [11] Piot et al. (2001), [12] Prats Mateu et al. (2016), [13] Ram et al. (2003), [14] Schulz and Baranska (2007), [15] Sun et al. (2012),  [16] Synytsya et al. (2003), [17] Trebolazabala et al. (2013), [18] Wiercigroch et al. (2017), [19] Wu et al. (2011). | | | | |

**References:**

**Agarwal UP, Ralph SA. 1997.** FT-Raman spectroscopy of wood: Identifying contributions of lignin and carbohydrate polymers in the spectrum of black spruce (*Picea mariana*). *Applied Spectroscopy* **51**: 1648–1655.

**Agarwal UP, McSweeny JD, Ralph SA. 2011.** FT–Raman investigation of milled-wood lignins: softwood, hardwood, and chemically modified black spruce lignins. *Journal of Wood Chemistry and Technology* **31**: 324–344.

**Agarwal UP. 2014.** 1064 nm FT-Raman spectroscopy for investigations of plant cell walls and other biomass materials. *Frontiers in Plant Science* **5**: 490.

**Gierlinger N, Sapei L, Paris O. 2008.** Insights into the chemical composition of *Equisetum hyemale* by high resolution Raman imaging. *Planta* **227**: 969–980.

**Himmelsbach DS, Akin DE. 1998.** Near-infrared Fourier-transform Raman spectroscopy of flax (*Linum usitatissimum* L.) stems. *Journal of Agricultural and Food Chemistry* **46**: 991–998.

**Larsen KL, Barsberg S. 2010.** Theoretical and Raman spectroscopic studies of phenolic lignin model monomers. *Journal of Physical Chemistry B* **114**: 8009–8021.

**Littlejohn GR, Mans JC, Parker D, Lind R, Perfect S, Seymour M, Smirnoff N, Love J, Moger J. 2015.** *In vivo* chemical and structural analysis of plant cuticular waxes using stimulated Raman scattering microscopy. *Plant Physiology* **168**: 18–28.

**Lupoi JS, Smith EA. 2012.** Characterization of woody and herbaceous biomasses lignin composition with 1064 nm dispersive multichannel Raman spectroscopy. *Applied Spectroscopy* **66**: 903–910.

**Lupoi JS, Singh S, Parthasarathi R, Simmons BA, Henry RJ. 2015.** Recent innovations in analytical methods for the qualitative and quantitative assessment of lignin. *Renewable and Sustainable Energy Reviews* **49**: 871–906.

**Ma JF, Zhou X, Ma J, Ji Z, Zhang X, Xu F. 2014.** Raman microspectroscopy imaging study on topochemical correlation between lignin and hydroxycinnamic acids in *Miscanthus sinensis*. *Microscopy and Microanalysis* **20**: 956–963.

**Piot O, Autran J, Manfait M. 2001.** Investigation by confocal Raman microspectroscopy of the molecular factors responsible for grain cohesion in the *Triticum aestivum* bread wheat. Role of the cell walls in the starchy endosperm. *Journal of Cereal Science* **34**: 191–205.

**Prats Mateu B, Hauser MT, Heredia A, Gierlinger N. 2016.** Waterproofing in *Arabidopsis*: Following phenolics and lipids *in situ* by confocal Raman microscopy. *Frontiers in Chemistry* **4**: 10.

**Ram MS, Dowell FE, Seitz LM. 2003.** FT-Raman spectra of unsoaked and NaOH-soaked wheat kernels, bran, and ferulic acid. *Cereal Chemistry* **80**: 188–192.

**Schulz H, Baranska M. 2007.** Identification and quantification of valuable plant substances by IR and Raman spectroscopy. *Vibrational Spectroscopy* **43**: 13–25.

**Sun L, Varanasi P, Yang F, Loque D, Simmons BA, Singh S. 2012.** Rapid determination of syringyl: guaiacyl ratios using FT-Raman spectroscopy. *Biotechnology and Bioengineering* **109**: 647–656.

**Synytsya A, Copikova J, Matejka P, Machovic V. 2003.** Fourier transform Raman and infrared spectroscopy of pectins. *Carbohydrate Polymers* **54**: 97–106.

**Trebolazabala J, Maguregui M, Morillas H, de Diego A, Madariaga JM. 2013.** Use of portable devices and confocal Raman spectrometers at different wavelength to obtain the spectral information of the main organic components in tomato (*Solanum lycopersicum*) fruits. *Spectrochimica Acta A Molecular and Biomolecular Spectroscopy* **105**: 391–399.

**Wiercigroch E, Szafraniec E, Czamara K, Pacia MZ, Majzner K, Kochan K, Kaczor A, Baranska M, Malek K. 2017.** Raman and infrared spectroscopy of carbohydrates: a review. *Spectrochimica Acta Part A Molecular and Biomolecular Spectroscopy* **185**: 317–335.

**Wu HW, Volponi JV, Oliver AE, Parikh AN, Simmons BA, Singh S. 2011.** *In vivo* lipidomics using single-cell Raman spectroscopy. *Proceedings of the National Academy of Sciences of the United States of America* **108**: 3809–3814.

Table Supplementary 3. Primer sequences and details.

| **Description** | **Primer** | **Primer sequence (5´- 3´ direction)** | **Gene ID** | **Product size (bp)** | **T_a_ (°C)** | **T_m_ (°C)** | **Amplification efficiency (%)** |
| --- | --- | --- | --- | --- | --- | --- | --- |
| PdNIP2-1 | **Forward** | GTAGGGGAATTGGCAGGCTT | XM_008804384.2 | 181 | 60 | 60.03 | 98.2 |
|  | **Reverse** | CCCCAGATAGAGTGCCCAAC |  |  |  | 59.82 |  |
| PdNIP2-2 | **Forward** | TAGCGACAACAGCCGGATTT | XM_008785804.2 | 146 | 60 | 60.04 | 98.8 |
|  | **Reverse** | GAAATGCCTCAACACAGCG |  |  |  | 60.11 |  |
| Actin | **Forward** | CTTTGGATCTCTCGCCCTCC | XM_008778129.2 | 148 | 60 | 59.89 | 97.5 |
|  | **Reverse** | CCAGCCTTCACCATTCCAGT |  |  |  | 59.96 |  |

Table Supplementary 4. Gene expression stability as evaluated by the BestKeeper tool.

| **gene** | *Actin* | *PdNIP2-1* | *PdNIP2-2* |
| --- | --- | --- | --- |
| **average (Ct)** | 21.66 | 26.91 | 24.31 |
| **SD (± Ct)** | 0.38 | 0.70 | 0.46 |
| **CV (% Ct)** | 1.76 | 2.60 | 1.88 |
| **SD (± fold change)** | 1.29 | 1.61 | 1.36 |
| **coefficient of correlation** | 0.97 | 0.94 | 0.85 |
| **Power of HKG (± fold change)** | 1.73 | 2.56 | 1.81 |

The samples of cDNA were diluted (10-times) prior to qPCR amplification. Cycle threshold value = Ct, HKG = housekeeping gene, SD = standard deviation, CV = coefficient of variation.
